# KIT-6 supported PhAA-Pd complex as a sustainable nanocatalyst for C–O coupling reactions

**DOI:** 10.1038/s41598-025-90824-4

**Published:** 2025-04-11

**Authors:** Abdulrahman A. Almehizia, Munthar Kadhim Abosaoda, Anjan Kumar, Vicky Jain, Suhas Ballal, Abhayveer Singh, Mamata Chahar, Suman Saini, Kamal Kant Joshi, Abhinav Kumar

**Affiliations:** 1 Department of Pharmaceutical Chemistry, College of Pharmacy, King Saud University, PO Box 2457, 11451 Riyadh, Saudi Arabia; 2 College of Pharmacy, The Islamic University, Najaf, Iraq; 3 College of Pharmacy, The Islamic University of Al Diwaniyah, Al Diwaniyah, Iraq; 4 Department of Electronics and Communication Engineering, GLA University, Mathura, 281406 India; 5 Department of Chemistry, Faculty of Science, Marwadi University Research Center, Marwadi University, Rajkot, Gujarat 360003 India; 6 Department of Chemistry and Biochemistry, School of Sciences, JAIN (Deemed to be University), Bangalore, Karnataka India; 7 Centre for Research Impact and Outcome, Chitkara University Institute of Engineering and Technology, Chitkara University, Rajpura, Punjab 140401 India; 8 Department of Chemistry, NIMS Institute of Engineering and Technology, NIMS University, Rajasthan, Jaipur, India; 9 Department of Chemistry, Chandigarh Engineering College, Chandigarh Group of Colleges, Jhanjeri, Mohali, Punjab 140307 India; 10 Department of Allied Science, Graphic Era Hill University, Dehradun, Uttarakhand 248002 India; 11 Graphic Era Deemed to be University, Dehradun, Uttarakhand India; 12 Department of Computer Engineering, Michigan State University, East Lansing, 48823 USA

**Keywords:** KIT-6, C–O, Coupling, Mesoporous, Phenylalanine, Chemistry, Catalysis, Catalyst synthesis

## Abstract

A practical and efficient method for synthesizing C–O cross-coupling has been established using a Pd complex anchored on mesoporous KIT-6. This design showcases exceptional efficiency, recoverability, and thermal stability. The synthesized mesostructure underwent comprehensive characterization through techniques such as FT-IR, SEM, XRD, EDX, BET, ICP, and TGA analyses. This catalyst was then successfully applied in C–O cross-coupling reactions. The approach offers several advantages, including rapid reaction times, high yields, excellent product purity, simplicity, environmental friendliness, and straightforward work-up procedures. Importantly, the durable nanohybrid catalyst exhibited no metal leaching and retained its catalytic performance across multiple cycles of use.

## Introduction

Recovering and reusing nanocatalysts presents a significant challenge in contemporary research, mainly due to the high cost of these catalysts or the substantial value of the resulting products, both economically and medicinally. In advancing green chemistry principles, catalysis plays a critical role. The development of eco-friendly and sustainable catalytic methods hinges largely on improving the performance and efficiency of catalysts. Transition-metal-based catalysts are particularly suitable for organic transformations^1,2^. However, their widespread application also brings challenges, such as the leaching of expensive or toxic metals into desirable products, which remains a notable drawback when using heterogeneous metal-based catalysts^3,4^. The reusability of nanocatalysts remains a critical challenge in contemporary research^5,6^. Developing and fabricating heterogeneous nanocatalysts with the capacity for recycling and reuse has emerged as a key pathway in advancing sustainable and innovative catalytic strategies^7^. To address issues related to catalyst recovery and reusability, the immobilization of nanocatalysts onto heterogeneous supports has been proposed^8,9^. This strategy combines the benefits of both homogeneous and heterogeneous catalysis^10^. In recent years, heterogeneous systems have gained significant attention across various domains within the academic and industrial scientific communities, including industrial and green chemistry, due to their versatile applications at the interface between homogeneous and heterogeneous catalysts^11,12^. Over the past few decades, considerable efforts have been directed toward developing nanocatalysts by anchoring homogeneous precursors onto nano heterogeneous supports such as metal oxides, carbon materials, ionic liquids, polymers, silica gel, USY zeolites, alumina, and mesoporous silica (e.g., MCM-48, and MCM-41)^13,14^. This approach has become a pivotal strategy for creating advanced heterogeneous catalysts for chemical processes. Among the numerous catalyst supports available, mesoporous silica nanoparticles have emerged as particularly appealing due to their superior characteristics compared to homogeneous counterparts. These nanoparticles can be easily regenerated and reused after activation, making the overall process more economically sustainable. Notably, mesoporous silica KIT-6 has garnered special interest from researchers because of its exceptionally high surface area and outstanding efficiency. KIT-6 is utilized in a broad range of applications, including light harvesting, adsorption, sensing, catalysis, and gas storage. Additionally, its robust silica framework allows it to withstand high-pressure and high-temperature conditions, providing distinct advantages over other support materials^15–17^.

Carbon-based chemistry forms the foundation of numerous fascinating natural processes^18,19^. Although carbon atoms are generally unreactive with one another due to their elemental stability, C–O cross-coupling reactions provide a unique exception^20^. In these reactions, carbon atoms are brought into close contact with a palladium (Pd) atom, which serves as a catalyst to trigger the chemical transformation^2,21^. These Pd-catalyzed cross-coupling reactions play a pivotal role in chemical synthesis, constituting roughly one-fourth of all reactions performed within the pharmaceutical industry^22–24^. C–O coupling reactions play a crucial role in diverse applications, such as the synthesis of liquid crystal materials, hydrocarbons, pharmaceuticals, natural products, and advanced materials^25^. These reactions have emerged as a highly effective approach for the development of complex organic compounds, natural products, advanced materials, and biologically active compounds^26,27^.

Building on our ongoing research, this study presents the synthesis and characterization of KIT-6/n-Pr/PhAA-Pd, a highly efficient, stable, and reusable nanostructured catalyst designed to facilitate C–O coupling reactions.

## Experimental

### Chemicals and instrumentation

All the materials required for synthesizing the nanocatalyst, conducting the C-O coupling reaction, and obtaining the necessary reagents and solvents were procured from Merck or Fluka chemical companies. Infrared spectra of the samples were analyzed using a Bruker VERTEX 80 v model with KBr pellets in the range of 400–4000 cm^−1^. Nitrogen physisorption measurements were carried out at 77 K using a Quantachrome NOVA 4200e model. Before analysis, the samples were degassed and dried for 4 h at 90 °C under vacuum. The surface areas were calculated using the BET equation, while the average pore diameter and pore volume were determined from the nitrogen desorption branch. X-ray diffraction (XRD) measurements were performed with a PANalytical X’Pert PRO instrument. The material’s microstructure was characterized using both a scanning electron microscope (Zeiss Sigma VP model) and a transmission electron microscope (Zeiss EM10C-100 kV model). Energy-dispersive X-ray spectroscopy (EDX) analysis was conducted on a JEOL JEM-2010 instrument. Analytical thin-layer chromatography was performed with Merck silica gel GF254 plates. Proton nuclear magnetic resonance (^1^H NMR) (DMSO-d_6_, 400 MHz) spectra were recorded using BRUKER AVANCE instruments, employing DMSO-d_6_ as the solvent. Inductively coupled plasma (ICP) analysis was performed using a PerkinElmer LBKFB model. Thermogravimetric analysis (TGA) was conducted with an SDT Q600 V20.9 Build 20 model, and the superparamagnetic properties of the catalyst were assessed through vibrating sample magnetometry (VSM; MDKFD).

### Preparation of KIT-6/n-Pr/PhAA-Pd

**Fig. 1:**
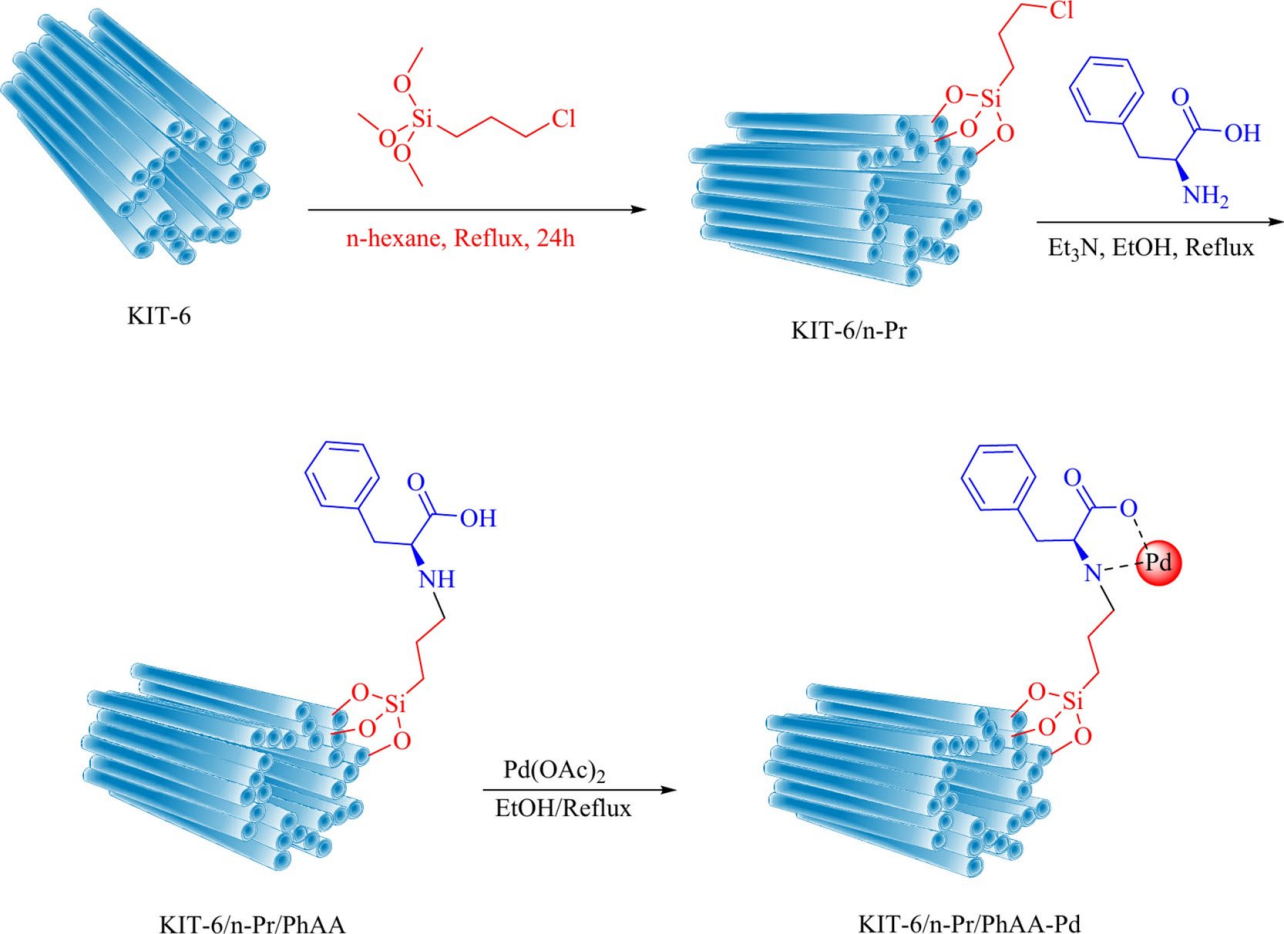
Preparation of KIT-6/n-Pr/PhAA-Pd.

The synthesis of mesoporous KIT-6 material was carried out using P123 as a structure-directing agent and n-butanol as a co-solvent within an acidic medium. The process began by dissolving 12 g of Pluronic P123 into 200 g of distilled water and 17 g of a 35% HCl solution. Following this, 8.0 g of n-butanol were added, and the mixture was stirred thoroughly at 30 °C. Subsequently, 15.0 g of TEOS were introduced, and the stirring continued for 24 h. After this step, the suspension was left to settle undisturbed at the same temperature. The solid product obtained was directly filtered without any washing and dried at 50 °C for 24 h. To finalize the synthesis, the template was removed by calcining the silica at 550 °C for 7 h. In the subsequent stage, a mixture of 3-chloropropyltrimethoxysilane (3.5 g) in n-hexane (40 mL) was slowly added dropwise into the precipitated material from the previous step and refluxed at 80 °C for 24 h. The resulting product was then dried under vacuum at 67 °C for 6 h. Afterward, phenylalanine (1 g), ethanol (50 mL), and triethylamine (Et_3_N, 1.7 mL) were incorporated into the reaction medium under reflux conditions for another 24 h. The product was subsequently dried in an oven for 12 h. In the final stage, Pd(OAc)_2_ (3 mmol) dissolved in absolute ethanol (30 mL) was added to 1 g of KIT-6/n-Pr/PhAA compound. This mixture was placed under reflux for 24 h and then dried at 60 °C to yield a novel catalyst (Fig. 1).

### General procedure for C–O coupling reaction

**Fig. 2:**
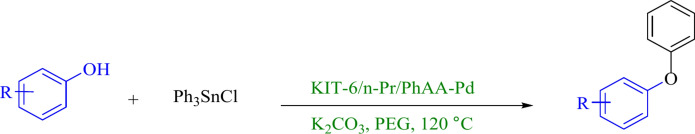
Synthesis of C–O bond catalyzed by KIT-6/n-Pr/PhAA-Pd.

A mixture containing triphenyltin chloride (1.2 mmol), phenol (1 mmol), and K_2_CO_3_ (1.1 mmol) was stirred in PEG at 120 ℃, with KIT-6/n-Pr/PhAA-Pd (30 mg) as the catalyst. The reaction progress was monitored using thin-layer chromatography (TLC) with hexane as the solvent. After completion, the KIT-6/n-Pr/PhAA-Pd catalyst was separated by filtration and washed with ethyl acetate and water, using sodium sulfate to absorb any moisture. Finally, the organic solvent was removed through evaporation to obtain the purified products (see Fig. 2).

### Selected NMR data

**1-Bromo-4-phenoxybenzene:** ^1^H NMR (400 MHz, DMSO): δ_H_ = 7.25–7.49 (m, 9H) ppm.

**Oxydibenzene:** ^1^H NMR (400 MHz, DMSO): δ_H_ = 7.57–7.65 (m, 10H) ppm.

**1-Nitro-4-phenoxybenzene:** ^1^H NMR (400 MHz, DMSO): δ_H_ = 7.10–7.23 (m, 9H), ppm.

## Results and discussion

### Catalyst characterization

**Fig. 3:**
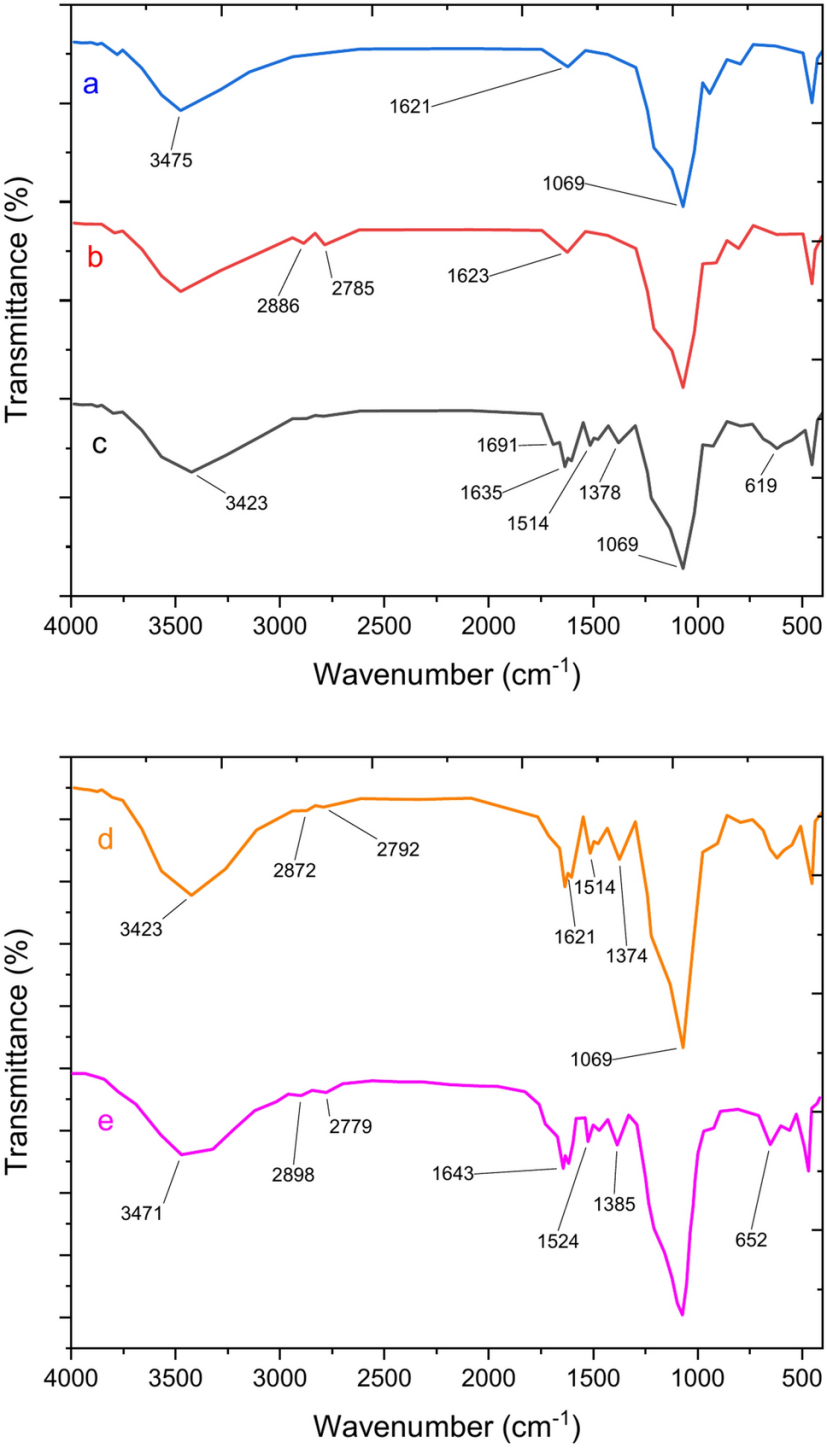
FT-IR analysis of (**a**) KIT-6 (**b**) KIT-6/n-Pr (**c**) KIT-6/n-Pr/PhAA, (**d**) KIT-6/n-Pr/PhAA-Pd and (**e**) recovered catalyst.

Figure 3 presents the FTIR spectra of KIT-6, KIT-6/n-Pr, KIT-6/n-Pr/PhAA, and KIT-6/n-Pr/PhAA-Pd materials. In Fig. 3a, a prominent, broad peak centered at 1069 cm⁻^1^ highlights the siloxane band. The FTIR spectrum of KIT-6/n-Pr in Fig. 3b displays characteristic bands around 2785 and 2886 cm⁻^1^, attributed to aliphatic C-H stretching vibrations associated with the alkyl chains. Figure 3c reveals peaks at 1375 and 1514 cm⁻^1^, corresponding to N–H bending vibrations of the amine groups, alongside a peak at 3423 cm⁻^1^ linked to the acidic group within the phenylalanine structure. The subtle variation in band intensity observed in Fig. 3d is attributed to the incorporation of Pd into the internal channels of the KIT-6/n-Pr/PhAA nanoparticles. Figure 3e presents the FT-IR spectrum of the recycled nanocatalyst, showing no changes in the FT-IR of KIT-6/n-Pr/PhAA-Pd after recovery, confirming its stability.

**Fig. 4:**
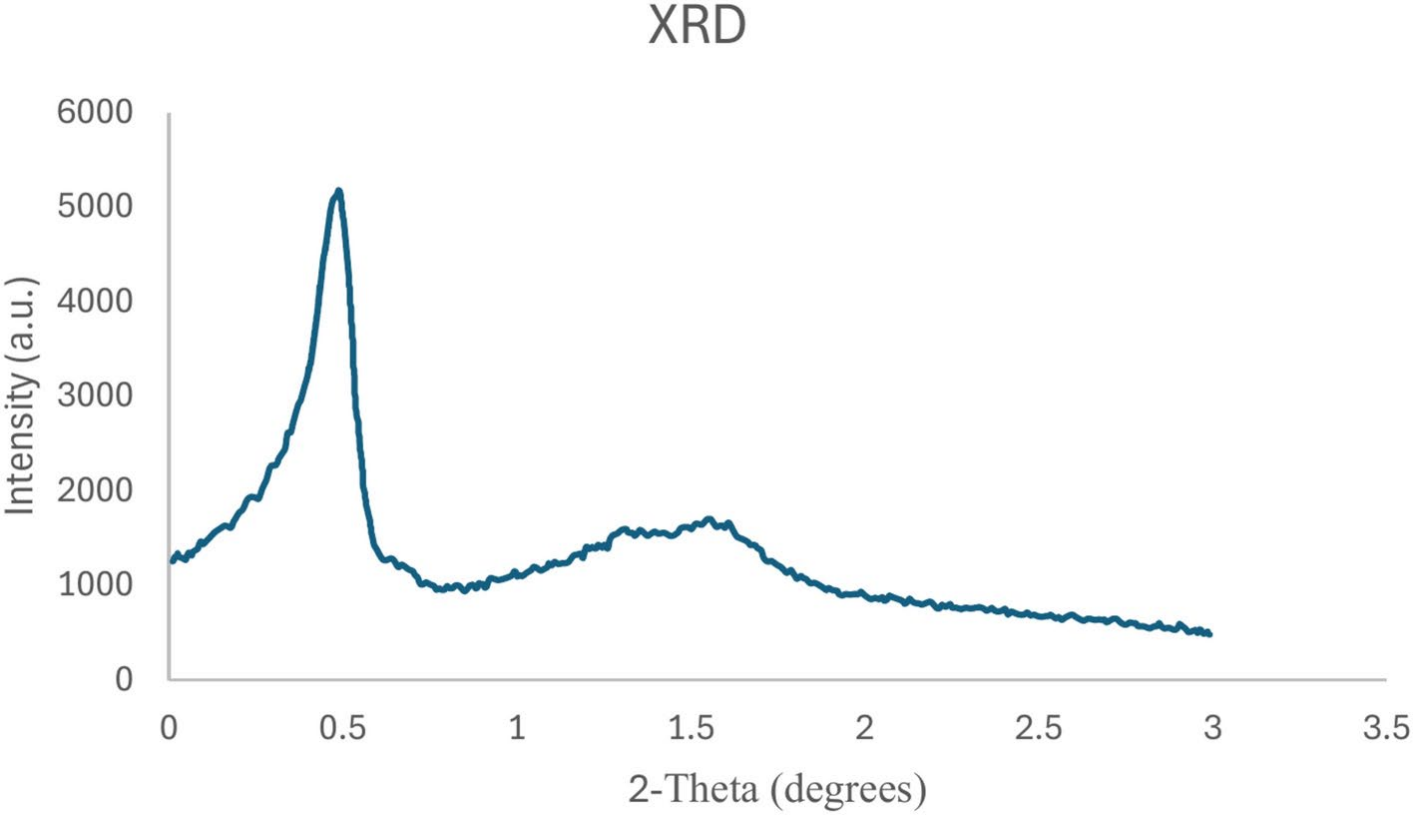
XRD spectrum of KIT-6/n-Pr/PhAA-Pd.

The low-angle powder X-ray diffraction (XRD) pattern of KIT-6/n-Pr/PhAA-Pd is illustrated in Fig. 4. The XRD patterns reveal a prominent peak associated with the pore family at the (100) reflection, along with two weaker (110) and (200) reflections corresponding to the family planes. These observations signify a well-defined hexagonal mesoscopic structure.

**Fig. 5:**
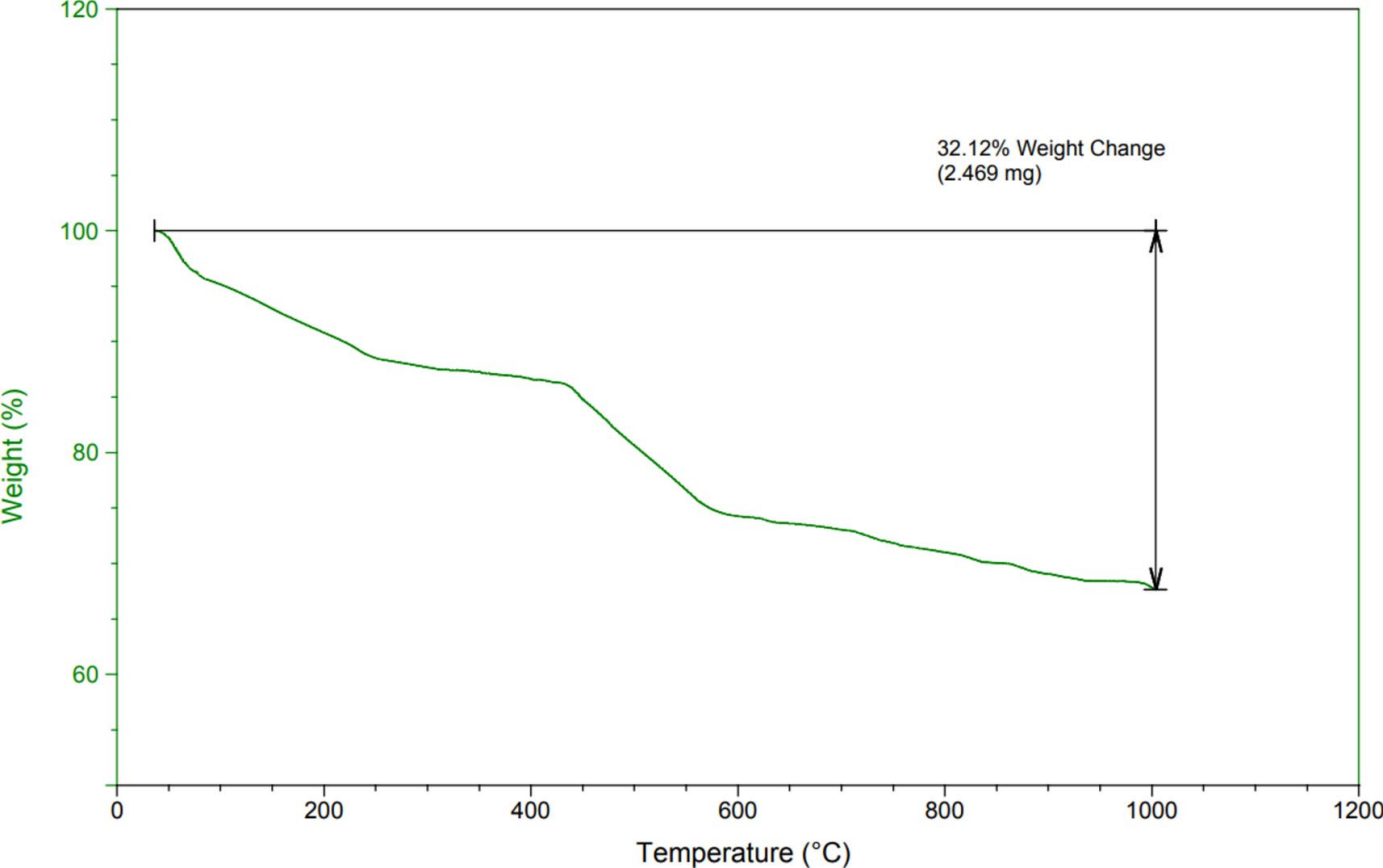
The TGA curves of KIT-6/n-Pr/PhAA-Pd.

The thermogravimetric analysis presented in Fig. 5 for KIT-6/n-Pr/PhAA-Pd nanoparticles highlights a two-step weight reduction process leading to an overall loss of 32.12% during the analysis. The first step, occurring below 250 °C, shows an 8% weight loss, attributed to the evaporation of residual moisture in KIT-6/n-Pr/PhAA-Pd. The second step, between 250°C and 600 °C, accounts for a 20% weight reduction caused by the degradation and decomposition of organic moieties. These observations confirm the successful incorporation of acid functional groups into the channels of KIT-6. At temperatures ranging from 600 °C to 1000 °C, an additional 4.12% weight loss is observed, attributed to the loss of surface hydroxyl groups.

**Fig. 6:**
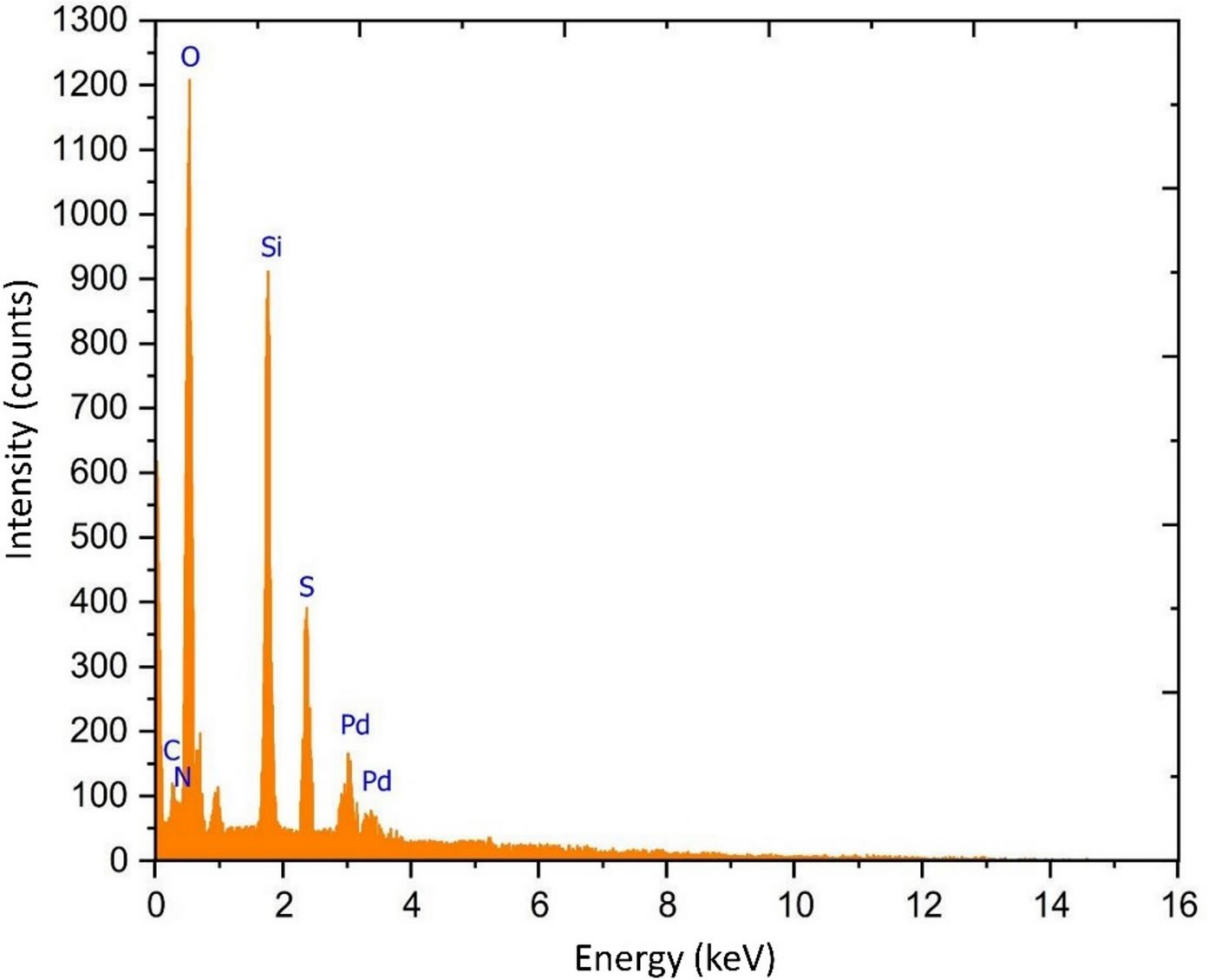
EDS spectrum of KIT-6/n-Pr/PhAA-Pd.

In a separate analysis, illustrated in Fig. 6, the EDX spectrum verified the presence of N, C, O, S, Si, and Pd elements in the synthesized material, confirming the successful preparation of KIT-6/n-Pr/PhAA-Pd. The detection of Pd peaks within the spectrum further validated the incorporation of Pd into the KIT-6/n-Pr/PhAA framework, affirming the effective synthesis of the catalyst.

**Fig. 7:**
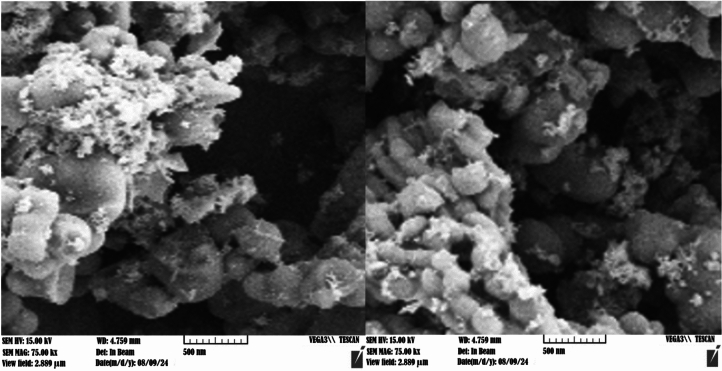
SEM spectrum of KIT-6/n-Pr/PhAA-Pd.

The particle size and morphology of KIT-6/n-Pr/PhAA-Pd were analyzed using scanning electron microscopy (SEM). Figure 7 illustrates the SEM images of this catalyst, revealing uniform and nearly spherical nanoparticles. The SEM analysis confirmed that the catalyst particles were successfully prepared at the nanometer scale.

**Fig. 8:**
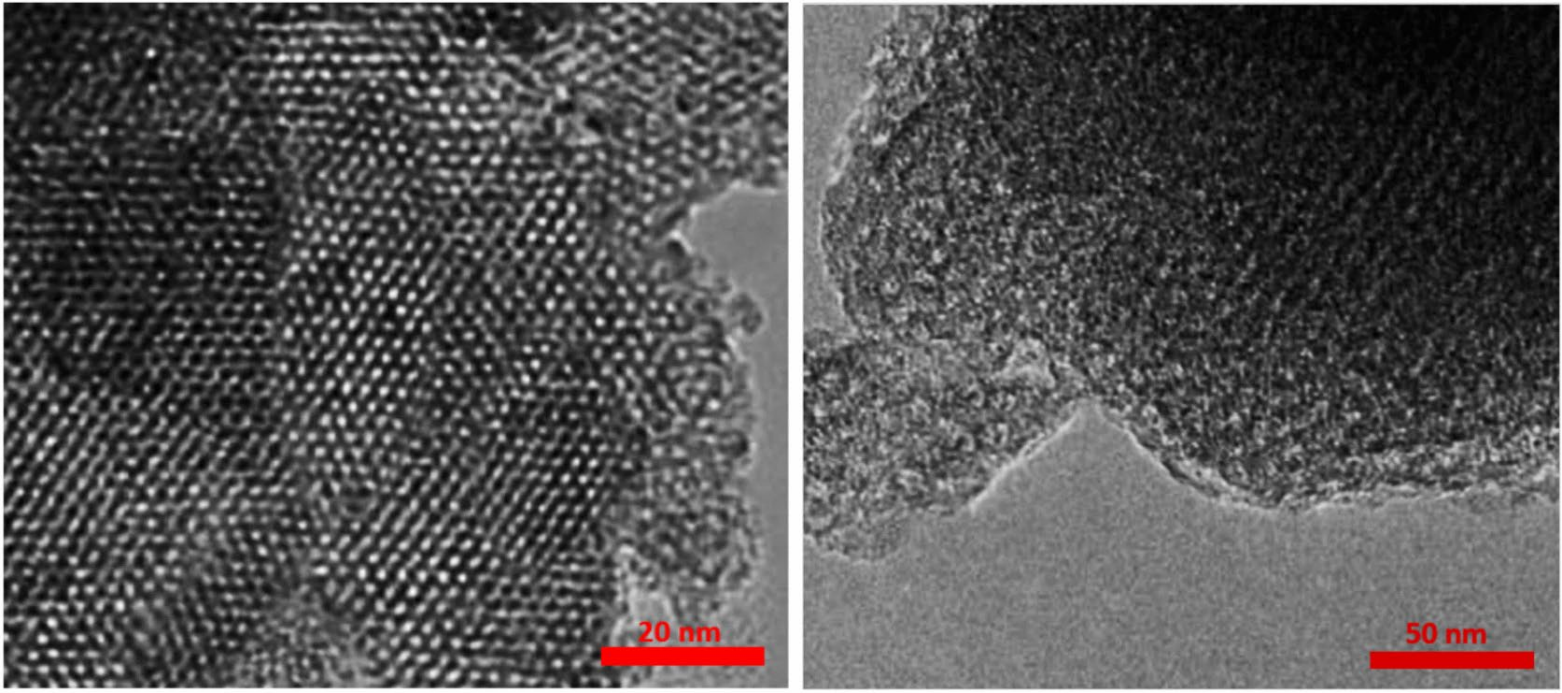
TEM spectrum of KIT-6/n-Pr/PhAA-Pd.

TEM micrographs offer precise insights into the size and surface morphology of particles. Figure 8 presents TEM images of the prepared sample under varying magnifications. The hexagonal pore structure, uniform particle size, and well-organized pore arrangement are distinctly visible, confirming the three-dimensional, long-range mesoporous ordering within this catalyst. Based on these findings, the morphology remains largely unchanged following the incorporation of Pd complex groups into KIT-6.

The ICP method was utilized to assess Palladium concentrations in the original catalyst and to analyze Palladium leaching after the recycling process. Findings showed that the Palladium content in the fresh and recycled catalysts was 2.7 × 10⁻^4^ and 2.5 × 10⁻^4^ mol g⁻^1^, respectively, demonstrating negligible leaching of Palladium from the KIT-6/n-Pr/PhAA-Pd structure.

**Fig. 9:**
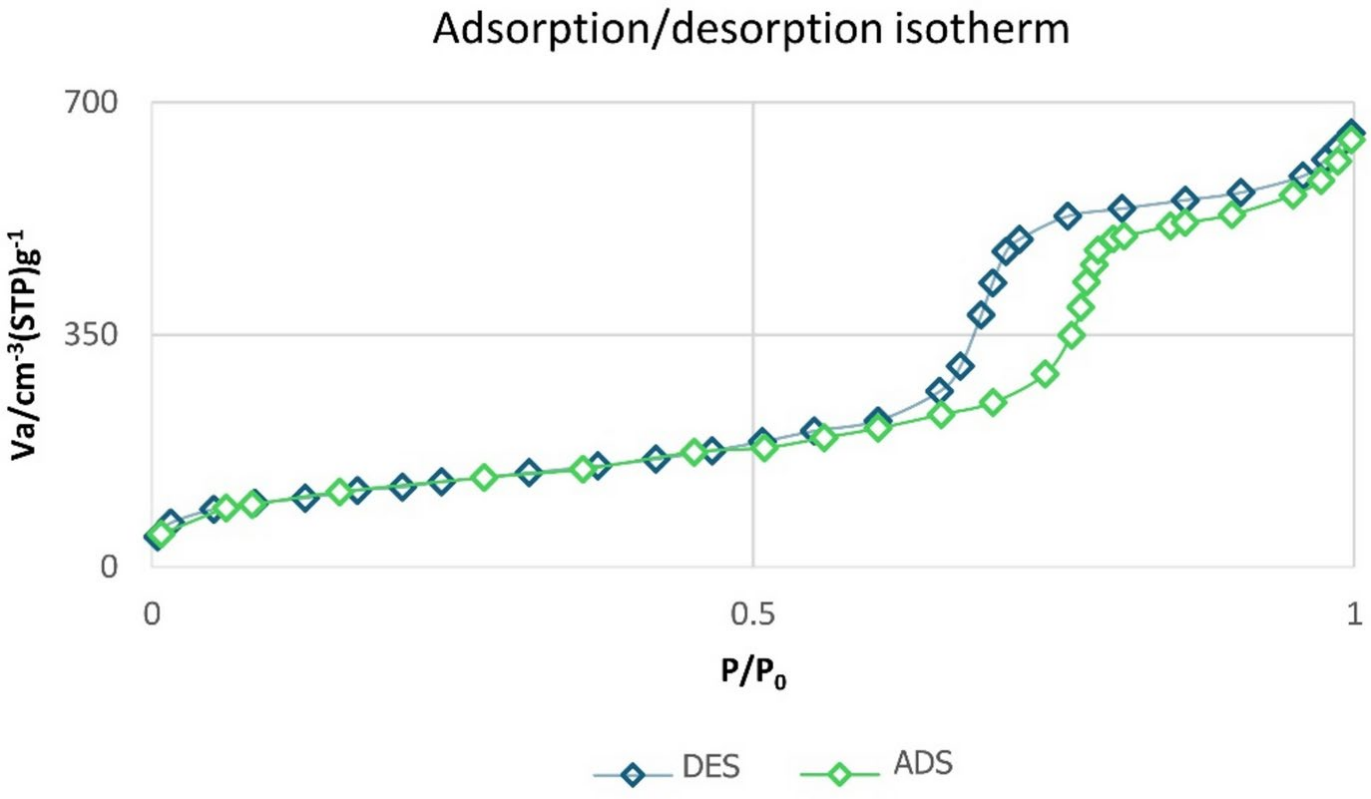
N_2_-adsorption isotherms of KIT-6/n-Pr/PhAA-Pd.

The pore size and surface area distribution of mesoporous silica KIT-6 were analyzed using N_2_ adsorption–desorption isotherms, as shown in Fig. 9. Based on these isotherms, the measured surface area of mesoporous silica KIT-6/n-Pr/PhAA-Pd was determined to be 412.77 m^2^/g. Additionally, the mean pore volumes were calculated using the BJH technique, with the total pore volume found to be 1.22 cm^3^/g.

## Catalytic studies

The subsequent step involved examining the catalytic properties of KIT-6/n-Pr/PhAA-Pd in various organic reactions, with a particular focus on the C-O coupling reaction. The catalytic performance of KIT-6/n-Pr/PhAA-Pd was assessed for C-O bond formation using triphenyl-tin chloride as a model reaction. Initially, the reaction between Ph_3_SnCl and phenol was examined under various conditions, including changes in bases, solvents, and the amount of KIT-6/n-Pr/PhAA-Pd catalyst, as detailed in Table 1. The effect of catalyst concentration was further evaluated in PEG as the solvent, using triphenyl-tin chloride at 120 °C without any catalyst present. After 24 h, no biphenyl product was formed under these conditions. However, the reaction’s efficiency improved significantly when adjusting the catalyst amount, with 0.03 g of KIT-6/n-Pr/PhAA-Pd identified as the optimal quantity. Employing different solvents, such as water (H_2_O), acetonitrile, PEG, ethanol (EtOH), or performing the reaction under solvent-free conditions, resulted in high product yields ranging from 41 to 98%. Among these options, PEG proved to be the most effective solvent. Based on these results, the optimized reaction conditions involve using 30 mg of KIT-6/n-Pr/PhAA-Pd catalyst, 1 mmol of phenol, 1.2 mmol of triphenyl-tin chloride, and 1.1 mmol of K_2_CO_3_ as the base in PEG at 120 °C.

**Table 1:** Optimization of different parameters for the reaction of phenol with Ph_3_SnCl.


Entry	Cat. (g)	Solvent	Base	T (˚C)	T (min)	Yield (%)^a,b^
1	–	PEG	K_2_CO_3_	120	1 day	N. R
2	0.007	PEG	K_2_CO_3_	120	60	41
3	0.01	PEG	K_2_CO_3_	120	60	84
4	0.02	PEG	K_2_CO_3_	120	60	91
5	0.03	PEG	K_2_CO_3_	120	60	98
6	0.035	PEG	K_2_CO_3_	120	60	98
7	0.03	H_2_O	K_2_CO_3_	120	60	42
8	0.03	Solvent-Free	K_2_CO_3_	120	60	78
9	0.03	CH_3_CN	K_2_CO_3_	120	60	79
10	0.03	EtOH	K_2_CO_3_	120	60	86
11	0.03	PEG	K_2_CO_3_	90	60	82
12	0.03	PEG	K_2_CO_3_	60	60	57
13	0.03	PEG	Cs_2_CO_3_	120	60	88
14	0.03	PEG	Na_2_CO_3_	120	60	80
15	0.03	PEG	KOH	120	60	43

^a^Reactions were run in 3 mL solvent with 1 mmol phenol, 1.2 mmol triphenyl-tin chloride, 30 mg of KIT-6/n-Pr/PhAA-Pd (catalyst), and 1.1 mmol base at 120 °C.

^b^Isolated yield.

Following the optimization of reaction conditions, the carbon–oxygen coupling reaction between various phenols and triphenyltin chloride (Ph_3_SnCl) was evaluated (Table 2). A range of phenols was tested in the coupling reaction with triphenyltin chloride, utilizing KIT-6/n-Pr/PhAA-Pd as the catalyst. Both electron-rich and electron-deficient phenols successfully underwent C–O coupling under mild conditions. The target products were achieved with efficiencies ranging from 91 to 98%.

**Table 2:** The reaction of phenol derivatives with Ph_3_SnCl.


Entry	Aryl halide	Product	Time (h)	Yield (%)^a,b^	TON	TOF (min^−1^)
1	Ph		1	98	122	122
2	3-MePh		1.5	90	112	74
3	4-MeOPh		2	95	119	59
4	3-MeOPh		1.5	92	115	76
5	4-MePh		2	94	117	58
6	3-BrPh		3	91	114	38
7	4-BrPh		4	85	106	26
8	4-NO_2_Ph		1.5	93	116	77
9	4-ClPh		1	95	118	118
10	2-Naph		3.5	93	116	33

^a^All reactions were carried out with ArOH (1 mmol), triphenyl-tin chloride (1.2 mmol), and K_2_CO_3_ (1.1 mmol) in the presence of the 30 mg of KIT-6/n-Pr/PhAA-Pd catalyst in 3 mL of PEG-400 at 120 ℃

^b^Isolated yield.

**Fig. 10:**
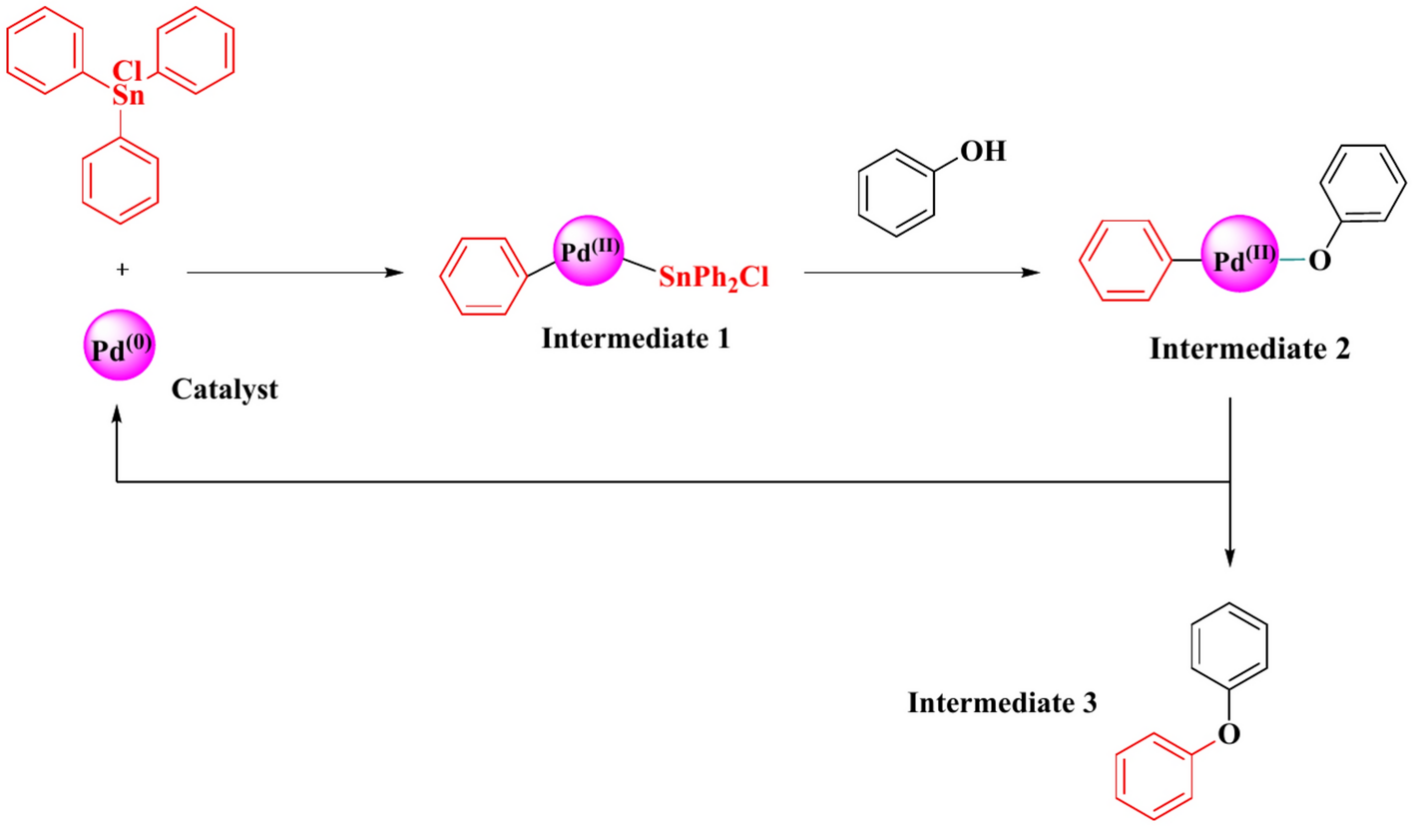
Proposed mechanism for C–O coupling.

The proposed mechanism for the Carbon–Oxygen cross-coupling reaction, illustrated in Fig. 10 and grounded in previous research, consists of multiple stages. Initially, Ph_3_SnCl undergoes oxidative addition with Pd, resulting in the formation of intermediate 1. Following this, intermediate 1 reacts with phenol to produce intermediate 2. This then proceeds to the final step, wherein reductive elimination occurs to form the ether product, accompanied by the release of the Palladium nanoparticle.

### Hot filtration

With optimal reaction conditions established, the heterogeneous nature of KIT-6/n-Pr/PhAA-Pd in the C-O coupling reaction was confirmed through a hot filtration experiment, using phenol as a model substrate. At the halfway mark of the reaction, the process was halted, yielding the product at 57%. In a parallel experiment, after reaching the same midpoint, the KIT-6/n-Pr/PhAA-Pd catalyst was removed from the reaction system via simple filtration. The reaction was then allowed to proceed without the nanocatalyst under identical conditions. Only minimal product conversion (< 3%) occurred after heating the catalyst-free mixture for an additional 30 min. These observations confirm the heterogeneous nature of the KIT-6/n-Pr/PhAA-Pd catalyst.

### Reusability of catalyst

**Fig. 11:**
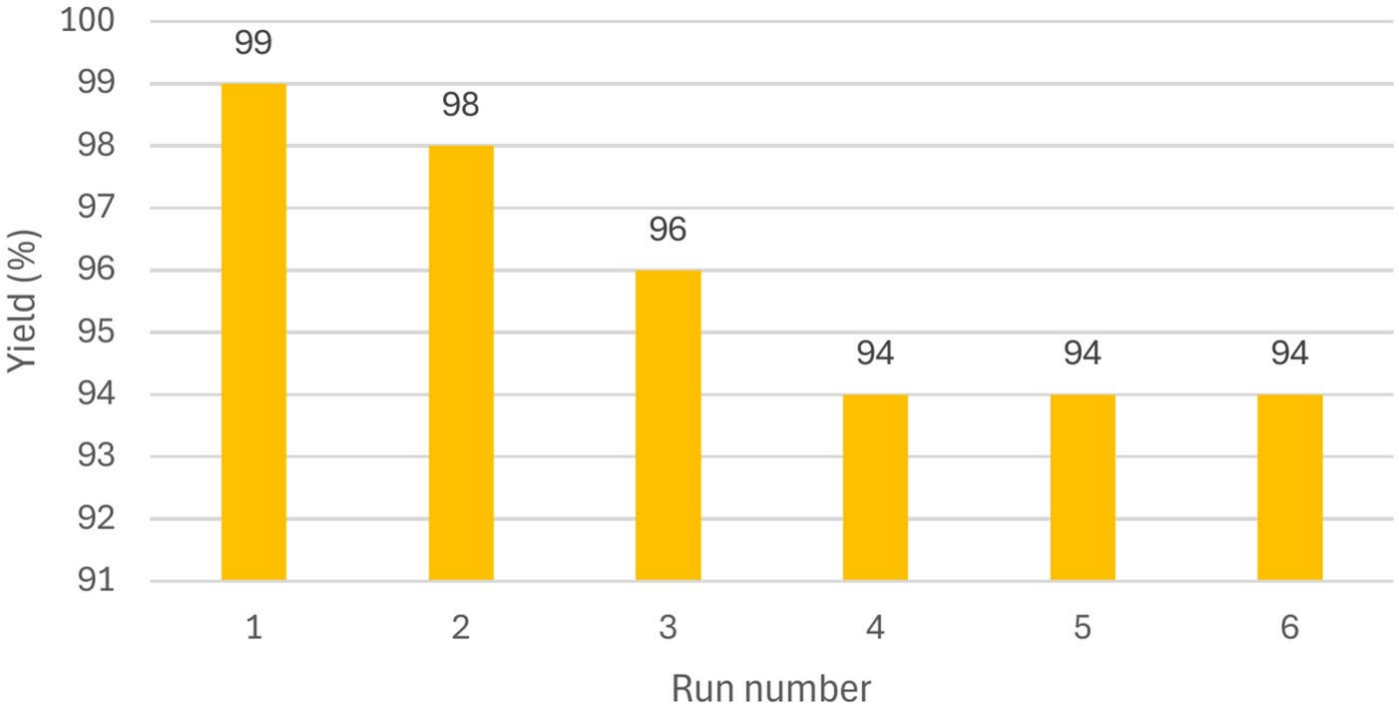
Reusability of KIT-6/n-Pr/PhAA-Pd in the C–O coupling reaction.

The recyclability of KIT-6/n-Pr/PhAA-Pd was evaluated in the reaction between phenol and Ph_3_SnCl to synthesize an oxydibenzene derivative. After completing the reaction, the catalyst was separated from the product and thoroughly washed with ethyl acetate. The findings indicated that the catalyst could be reused for up to six cycles with only a negligible loss in activity, as shown in Fig. 11. According to the process described in the experimental section, the as-prepared catalyst was easily recovered using filter paper. This excellent reusability may be attributed to the strong bond formed between the immobilized PhAA-Pd complex and KIT-6, which helps prevent Pd leaching, as depicted in Fig. 11.

## Conclusions

In conclusion, a novel and environmentally friendly KIT-6/n-Pr/PhAA-Pd catalyst was successfully prepared, with its synthesis validated through various analytical techniques, including FT-IR, VSM, XRD, ICP, TGA, EDX, TEM, and SEM. This study presented an innovative approach for the synthesis of diverse C-O coupling reactions, achieving high to excellent yields. A notable aspect of this research is the exceptional purity of the products obtained. Furthermore, the catalytic activity of this nanostructured material was explored as a recyclable system for C-O coupling reactions. Key advantages of this method include a straightforward work-up process, affordability and chemical stability of the reagents, ease of separation via filtration, reusability of the catalyst, and the use of readily available commercial reagents.

## Supplementary Information

Supplementary Information.

## Data Availability

The data produced or examined in this study is fully available within the published article and its supplementary information files.
