# Supplementary material for: KIT-6 supported PhAA-Pd complex as a sustainable nanocatalyst for C–O coupling reactions
Source: Sci Rep. 2025 Apr 11;15:12379. doi: 10.1038/s41598-025-90824-4 (PMC11985943; doi:10.1038/s41598-025-90824-4)
Supplement: Supplementary file 1 — Supplementary Information. [file 41598_2025_90824_MOESM1_ESM.docx]

**KIT-6 supported PhAA-Pd complex as a sustainable nanocatalyst for C-O coupling reactions**


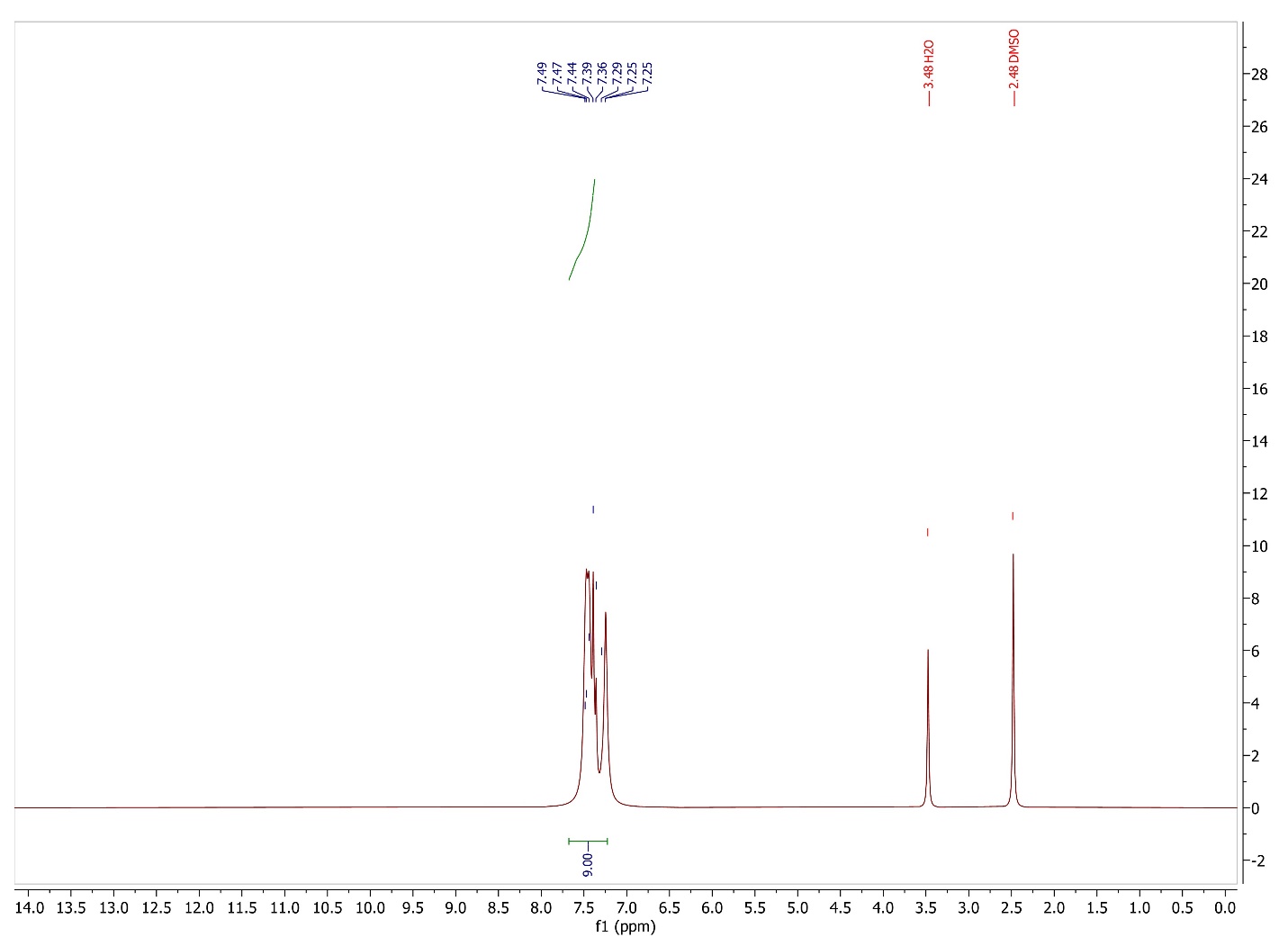


**S1.** **1-bromo-4-phenoxybenzene**

**S1. 1-bromo-4-phenoxybenzene:** ^1^H NMR (400 MHz, DMSO): δ_H_= 7.25-7.49 (m, 9H) ppm.


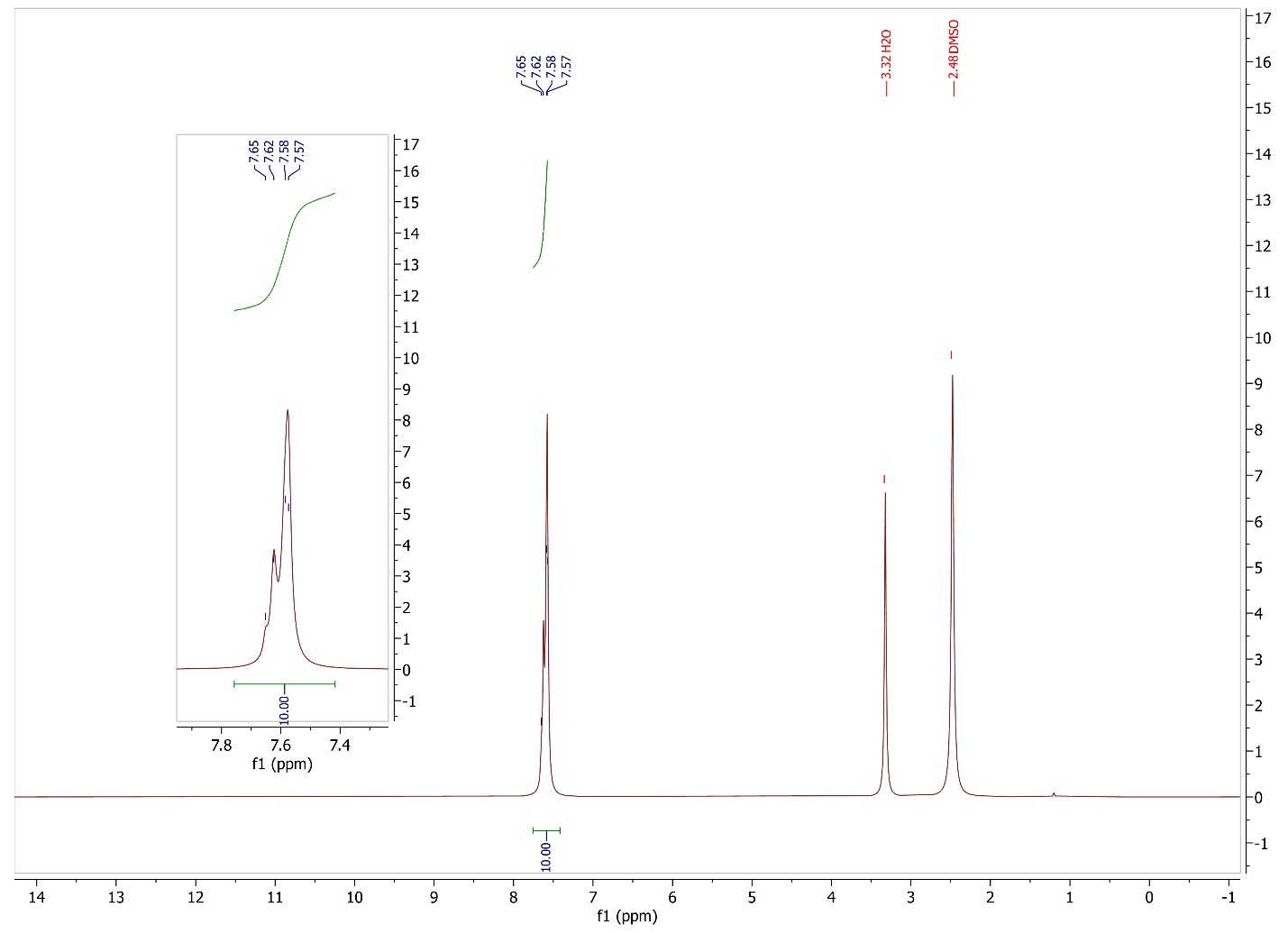


**S2.** **Oxydibenzene**

**S2. Oxydibenzene:** ^1^H NMR (400 MHz, DMSO): δ_H_= 7.57-7.65 (m, 10H) ppm.


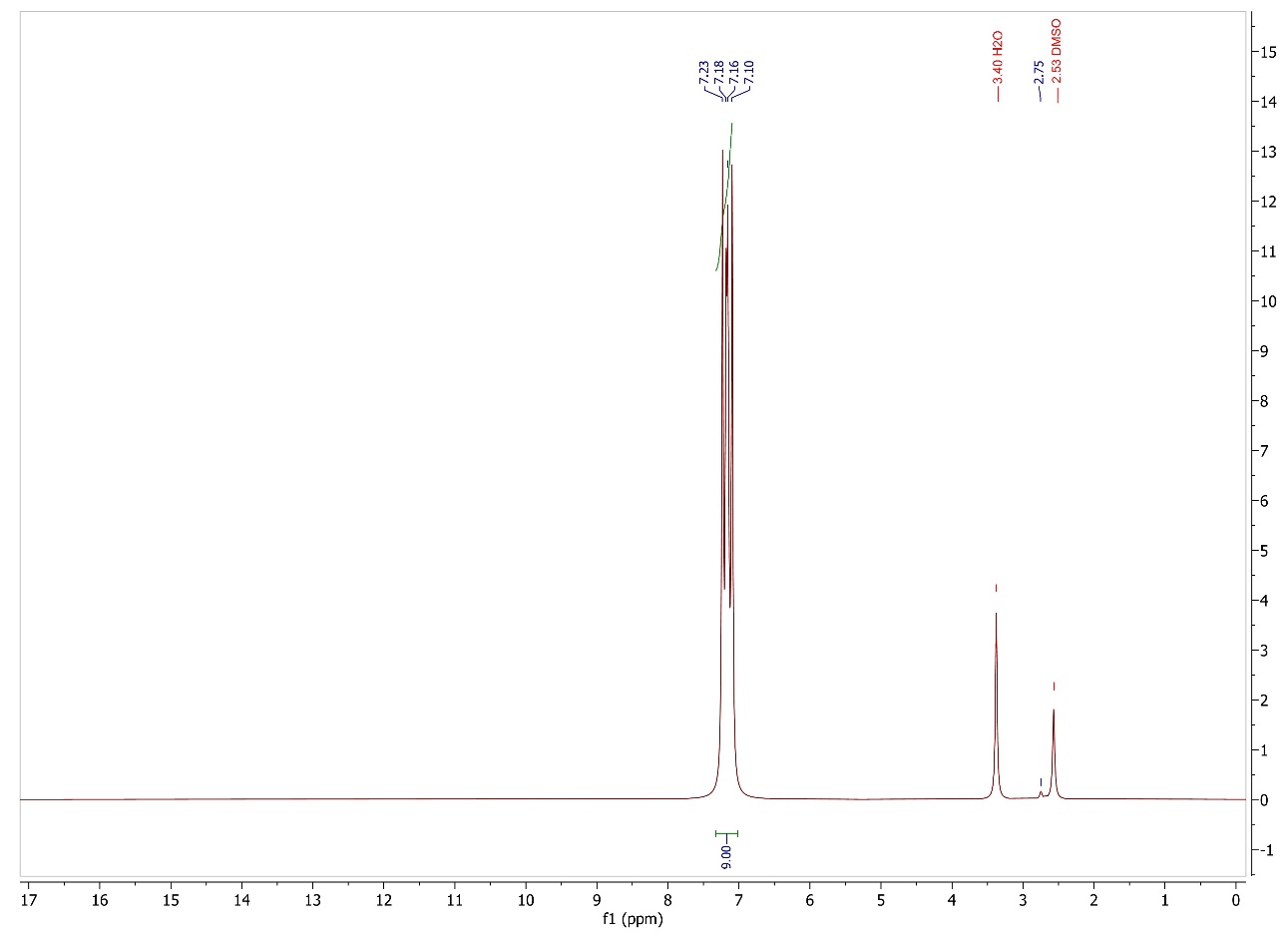


**S3.** **1-Nitro-4-phenoxybenzene**

**S3. 1-Nitro-4-phenoxybenzene:** ^1^H NMR (400 MHz, DMSO): δ_H_= 7.10-7.23 (m, 9H), ppm.
